# Retraction Note: GraphCovidNet: A graph neural network based model for detecting COVID-19 from CT scans and X-rays of chest

**DOI:** 10.1038/s41598-021-02469-8

**Published:** 2021-11-30

**Authors:** Pritam Saha, Debadyuti Mukherjee, Pawan Kumar Singh, Ali Ahmadian, Massimiliano Ferrara, Ram Sarkar

**Affiliations:** 1 Department of Electrical Engineering, Jadavpur University, Kolkata, 700032 India; 2 Department of Computer Science and Engineering, Jadavpur University, Kolkata, 700032 India; 3 Department of Information Technology, Jadavpur University, Kolkata, 700106 India; 4 Institute of IR 4.0, The National University of Malaysia, Bangi, 43600 UKM Selangor Malaysia; 5 School of Mathematical Sciences, College of Science and Technology, Wenzhou-Kean University, Wenzhou, China; 6 ICRIOS-The Invernizzi Centre for Research in Innovation, Organization, Strategy and Entrepreneurship, Department of Management and Technology, Bocconi University, Via Sarfatti, 25, 20136 Milan (MI), Italy

Retraction of: *Scientific Reports* https://doi.org/10.1038/s41598-021-87523-1, published online 15 April 2021

The Editors have retracted this Article.

The testing accuracy for the method reported in the Article was found to be over 99% for all datasets. However, it has been brought to the Editors' attention after the publication of the Article that the graph labels were included as node-level attributes. These attributes were then used by the classifier to predict the graph label, violating the testing procedure, and leading to an over-estimation of the model's classification accuracy.

Ali Ahmadian and Massimiliano Ferrara disagree with this retraction. Pritam Saha, Debadyuti Mukherjee, Pawan Kumar Singh and Ram Sarkar have not responded to correspondence from the Editors about this retraction.

